# Pediatric Infective Endocarditis Due to Streptococcus mitis: A Case Report

**DOI:** 10.7759/cureus.92161

**Published:** 2025-09-12

**Authors:** Raúl Romero Feregrino, Daniel O Pacheco Rosas, María J Carlos De la Torre

**Affiliations:** 1 Department of Pediatrics, Mexican Academy of Pediatrics, Mexico City, MEX; 2 Department of Pediatrics/Infectious Diseases Service, Centro Médico Nacional Siglo XXI, Mexico City, MEX; 3 Department of Infectious Disease, Hospital de Pediatría Centro Médico Nacional Siglo XXI, Mexico City, MEX

**Keywords:** infective endocarditis, microbiological diagnosis, pediatrics, septic pulmonary embolism, streptococcus mitis

## Abstract

Infective endocarditis in pediatrics is a rare but serious condition, even in immunocompetent children without congenital heart disease. *Streptococcus mitis*, a commensal inhabitant of the oral cavity, has emerged as a relevant etiological agent capable of causing invasive infections due to its strong endothelial adherence and biofilm formation. Accurate microbiological identification requires advanced tools such as matrix-assisted laser desorption ionization time-of-flight (MALDI-TOF) mass spectrometry, given its genetic similarity to *Streptococcus pneumoniae*. This case report presents a 12-year-old female patient with a history of a ventricular septal defect and persistent respiratory symptoms. After multiple ineffective treatments, an echocardiogram revealed cardiac vegetations consistent with IE. Blood cultures confirmed *Streptococcus mitis* infection, prompting targeted antibiotic therapy. The patient underwent surgical resection of pulmonary valve vegetations and closure of the ventricular septal defect. Chest computed tomography revealed an infectious pneumatocele, suggesting septic embolization. This case highlights the potential for pulmonary involvement secondary to right-sided endocarditis, even in the absence of traditional risk factors. Vegetations larger than 10 mm were associated with lower response to medical therapy and a higher risk of embolization, supporting the need for surgical intervention. *Streptococcus mitis* should be considered a potential pathogen in pediatric infective endocarditis, even in patients without comorbidities. Persistent fever, positive blood cultures, and atypical pulmonary findings warrant prompt echocardiographic evaluation. Accurate diagnosis and early treatment are essential to reduce complications and improve clinical outcomes.

## Introduction

Infective endocarditis (IE) in pediatrics is an uncommon condition but one associated with high morbidity and mortality, especially when diagnosis is delayed or systemic complications arise [1]. Although it is traditionally associated with congenital heart disease or intracardiac devices, its occurrence in immunocompetent pediatric patients without structural predisposing factors is not unheard of and presents significant diagnostic challenges [2].

One of the most frequent etiological agents of IE in children without congenital heart disease is the viridans group streptococci, among which *Streptococcus mitis* stands out as an opportunistic commensal of the oral cavity with significant pathogenic potential [3]. *Streptococcus mitis* belongs to the mitis group within the *Streptococcus* genus. It is characterized by its Gram-positive coccoid morphology, arrangement in short chains, production of alpha-hemolysis on blood agar, and high genetic homology with *Streptococcus pneumoniae* [4]. This phylogenetic proximity has led to diagnostic difficulties in microbiological typing, requiring molecular techniques such as 16S rRNA gene sequencing or matrix-assisted laser desorption ionization time-of-flight (MALDI-TOF) mass spectrometry for accurate identification [5].

*Streptococcus mitis* is commonly found as a colonizing bacterium in the oral cavity, gastrointestinal tract, and female reproductive tract, but it can cause invasive diseases such as endocarditis and meningitis. The prevalence of *Streptococcus mitis* infections has shown an upward trend in recent years [6].

Although pulmonary involvement in IE is rare, it can occur in the context of right-sided heart vegetations with septic embolization, particularly in infections caused by highly adherent bacteria such as *Streptococcus mitis*, which possess surface proteins (such as PcsB and pilus-like adhesins) that facilitate colonization of endothelium and cardiac valves [7]. Respiratory manifestations may include nodular infiltrates, hemoptysis, or pulmonary abscesses, findings that can be mistaken for primary respiratory infections [8].

In this context, we present the case of an immunocompetent pediatric patient diagnosed with IE due to *Streptococcus mitis* with pulmonary involvement, aiming to highlight the importance of accurate etiological diagnosis and clinical suspicion in the face of unusual respiratory presentations.

## Case presentation

A 12-year-old female patient with a history of ventricular septal defect (VSD) diagnosed in the neonatal period, under routine cardiologic follow-up. She presented with a four-month history of symptoms that began with fever, hemoptysis, and exertional dyspnea (on moderate effort), which did not initially limit daily activities. During the course of her illness, she was treated multiple times for upper respiratory tract infections with different antimicrobials, without clinical improvement.

She attended a routine cardiology consultation, during which a transthoracic echocardiogram revealed vegetations, leading to the presumptive diagnosis of IE.

Upon hospital admission, physical examination revealed fever, grade I dental caries in the left lower first molar, intercostal retractions, prolonged expiration, a grade III holosystolic murmur heard in multiple areas, punctiform lesions on the distal third of both legs and right hand, non-palpable and non-blanching with pressure.

The laboratory studies showed elevated CRP, leukocytosis, neutrophilia, lymphopenia, increased hemoglobin and hematocrit levels, as well as decreased platelet count (Table 1).

**Table 1:** Laboratory studies

Laboratory studies	Result	Reference range
C-reactive protein	92.74 mg/L	< 10 mg/L
White blood cells	7250 /µL	4.5-11.0 × 10^9^/µL
Neutrophils	5160 /µL	1.5-7.0 × 10^9^/µL
Lymphocytes	1720 /µL	3.0-9.5 × 10^9^/µL
Hemoglobin	20.4 g/dL	11.5-15.5 g/dL
Hematocrit	61.3%	36-46%
Platelets	120,000 /µL	150,000-450,000 /µL

Transesophageal echocardiogram revealed perimembranous VSD measuring 3.9 × 3.4 mm, three vegetations: one on the right coronary cusp associated with the VSD (8 × 4.7 mm) and two on the pulmonary valve (12 × 4 mm and 7.5 × 3 mm).

The patient meets the following modified Duke criteria: positive blood cultures for the causative organism, an echocardiogram demonstrating vegetations, fever >38°C, imaging evidence of embolic phenomena (pulmonary CT), underlying congenital heart disease, and a heart murmur with increased intensity. Based on these findings, a diagnosis of IE was made, and empiric treatment with ceftriaxone (100 mg/kg/d) was initiated. Blood cultures taken upon admission (three out of five sets) yielded *Streptococcus mitis*, leading to adjustment of treatment to gentamicin (6 mg/kg/d) and crystalline penicillin (400,000 IU/kg/d).

**Figure 1:**
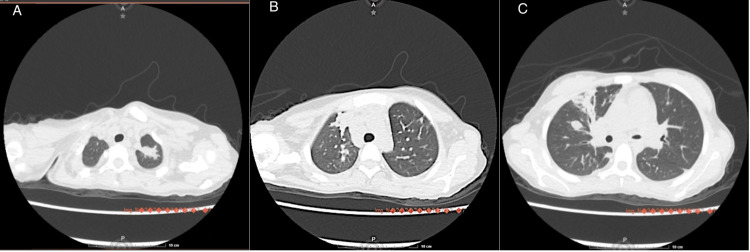
Computed tomography of the chest A) Axial slice at the level of the lung apices showing a parahilar lesion. B) Axial slice at the level of the middle lobe showing diffuse bilateral subsolid nodules. C) Consolidation of the posterior basal segment of the left lower lung lobe.

Given the echocardiographic findings, the patient underwent surgical wedge resection of the vegetations on the pulmonary valve and closure of the VSD using an autologous pericardial patch. Due to persistent respiratory symptoms, a high-resolution chest computed tomography (CT) was performed, revealing an infectious pneumatocele. Although no direct microbiological isolation from the lung was obtained, these findings were attributed to pulmonary involvement by *Streptococcus mitis* (Figure 1).

**Figure 2:**
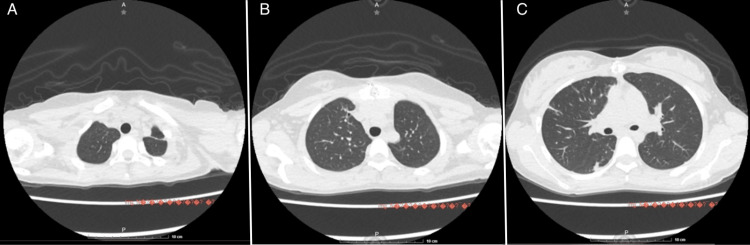
Follow-up computed tomography of the chest four weeks post-discharge A) Axial slice at the level of the lung apices showing improvement of previous lesions. B) Axial slice at the level of the middle lobe showing improvement of previous lesions. C) Improvement of the lesion in the posterior segment of the left lower lung lobe.

After two weeks of intravenous antibiotic therapy, a follow-up echocardiogram showed resolution of vegetations, and the patient demonstrated clinical improvement, including resolution of fever. She was transitioned to oral amoxicillin-clavulanic acid (dosage 150 mg/kg/d) for an additional four weeks with good clinical response. A follow-up chest CT at four weeks post-discharge showed significant improvement of the lesions (Figure 2).

## Discussion

IE in the pediatric population represents a significant diagnostic challenge, particularly in patients without a history of congenital heart disease or evident predisposing factors. This case highlights the ability of *Streptococcus mitis*, a microorganism typically regarded as a commensal, to act as an invasive pathogen and cause severe infections in immunocompetent children [1].

*Streptococcus mitis* belongs to the mitis group of viridans streptococci, and although its presence in the oral mucosa is commonly associated with transient bacteremia following dental procedures, it has also been linked to endocarditis - especially in the context of poor oral hygiene or disrupted mucosal barriers [2,3]. It is now understood that cumulative low-grade bacteremia, triggered by routine daily activities such as tooth brushing, flossing, and chewing, plays a more significant role in the pathogenesis of IE. This has led to increased emphasis on maintaining good oral hygiene [4].

*Streptococcus mitis* possesses adhesive capabilities and biofilm-forming potential, mediated by surface proteins such as PcsB and pilus-like adhesins, which contribute to its pathogenicity in IE. Genomic studies have shown that some *Streptococcus mitis* strains isolated from IE cases harbor virulence profiles similar to *Streptococcus pneumoniae*, including genes involved in immune evasion and tissue invasion [5,8].

In this case, the pulmonary manifestation resulted from right-sided cardiac vegetations (on the pulmonary valve) and subsequent septic embolization to the pulmonary circulation. While this pattern is more commonly seen in intravenous drug users or patients with central venous catheters, it can also occur in patients without these risk factors, particularly when the infecting organism has a high embolic potential [9,10]. The presence of cavitated pulmonary nodules or peripheral consolidations, as seen in our patient, should raise suspicion for pulmonary septic emboli, especially when accompanied by persistent fever and positive blood cultures [11].

Diagnosis of *Streptococcus mitis* IE requires accurate microbiological identification, as it can be easily mistaken for other alpha-hemolytic streptococci. MALDI-TOF mass spectrometry has proven to be a reliable tool for differentiating *Streptococcus mitis* from other viridans group species, with important therapeutic implications given that some strains demonstrate intermediate resistance to beta-lactam antibiotics [6,9]. In our case, the isolate was susceptible to penicillin and ceftriaxone, allowing for conventional treatment with good clinical outcomes.

Penicillin-sensitive streptococcal infections, such as in our patient, have a reported cure rate of over 95% [12]. Robbins et al. [12] found that while 100% of vegetations <10 mm responded to medical therapy alone, only 63% of vegetations >10 mm did, with the remainder requiring surgical intervention. They proposed that as bacterial colonies grow deeper within the vegetation, their metabolism and replication slow down, decreasing antibiotic effectiveness. In our case, one of the vegetations measured 12 × 4 mm, and a total of three vegetations were present, which prompted surgical management. Despite adequate treatment, vegetations larger than 10 mm embolize in 14% of cases, compared to only 1% in smaller vegetations [12-13].

This report underscores the importance of maintaining a high index of suspicion for IE in children with persistent fever, atypical pulmonary findings, and positive blood cultures - even in the absence of classic predisposing conditions. It also reinforces the need to consider *Streptococcus mitis* as a clinically relevant pathogen in this setting, rather than dismissing it as normal microbiota.

## Conclusions

*Streptococcus mitis* IE can occur in immunocompetent pediatric patients without prior heart disease, and should therefore be considered in the differential diagnosis of persistent fever with unusual pulmonary findings. Pulmonary involvement in the form of septic emboli may be a significant manifestation in cases of right-sided endocarditis, and early identification through imaging and microbiological studies can guide timely diagnosis and treatment. The use of advanced diagnostic tools such as MALDI-TOF mass spectrometry is essential for the accurate identification of *Streptococcus mitis*, distinguishing it from other viridans group streptococci, which have important therapeutic implications.
